# NLRP3 inflammasome activation by turbulent shear stress drives right ventricular outflow tract fibrosis in pulmonary regurgitation

**DOI:** 10.3389/fcvm.2025.1546581

**Published:** 2025-05-02

**Authors:** Qiang Fan, Yabo Wang, Dongyong Zhu, Qi An, Yunfei Ling

**Affiliations:** ^1^ Department of Cardiovascular Surgery, West China Hospital, Sichuan University, Chengdu, Sichuan, China; ^2^ Department of Radiology, Union Hospital, Tongji Medical College, Huazhong University of Science and Technology, Wuhan, Hubei, China

**Keywords:** pulmonary regurgitation, turbulent shear stress, right ventricle outflow track, fibrosis, NLRP3 inflammasome

## Abstract

**Objective:**

This study aimed to investigate the role of turbulent shear stress (TSS) induced by pulmonary regurgitation (PR) in driving right ventricular (RV) dysfunction, with a focus on NLRP3 inflammasome activation, inflammation, and fibrosis, particularly in the RV outflow tract (RVOT).

**Methods:**

Clinical data from 6 repaired tetralogy of Fallot (rTOF) patients with PR were analyzed using cardiac magnetic resonance (CMR) and computational fluid dynamics (CFD) to quantify TSS distribution. Human cardiomyocytes were cultured under static (SF), unidirectional (UF), or oscillatory flow (OF) conditions to simulate TSS. A rat PR model was established to assess RV remodeling over 4–12 weeks. NLRP3 expression, cytokine release, and fibrosis were evaluated via western blot, ELISA, and histology.

**Results:**

CFD revealed elevated turbulent kinetic energy (TKE) and TSS in the RVOT compared to inflow and apical regions (*P* = 0.001). *in vitro*, OF (15 dyn/cm^2^) activated NLRP3 inflammasome in cardiomyocytes, increasing NLRP3 (10-fold, *P* = 0.01) and caspase-1 (4-fold, *P* = 0.012), and elevating IL-1β (775.1 ± 9.4 vs. 658.4 ± 19.6 pg/ml, *P* = 0.03) and IL-18 (1,264.8 ± 10.7 vs. 1,038.6 ± 18.8 pg/ml, *P* = 0.022) levels compared to SF. *in vivo*, PR induced progressive RV dilation (RVEDVi: 7.4 ± 0.4–10.8 ± 0.6 ml/m^2^, *P* < 0.01) and reduced longitudinal strain (45.6 ± 2.5–19.1 ± 0.5 s^−1^, *P* < 0.01), and RVOT-predominant NLRP3 expression (12 weeks: 0.07 ± 0.02 vs. 0.005 ± 0.001 in controls, *p* < 0.001) and fibrosis (33.9 ± 4.8% vs. 12.8 ± 3.2% in control, *p* < 0.01).

**Conclusion:**

PR-induced TSS in the RVOT activates the NLRP3 inflammasome in cardiomyocytes, triggering inflammation and fibrosis that drive regional RV dysfunction. Quantifying TSS may serve as an early biomarker for subclinical RV injury, while targeting NLRP3 signaling could offer a therapeutic strategy to mitigate fibrosis in PR patients.

## 1 Introduction

Pulmonary regurgitation (PR) is a common sequela following surgical repair of congenital heart defects such as tetralogy of Fallot (TOF) (1), with 25%–50% of patients developing significant RV fibrosis more than a decade post-repair (2, 3). While chronic volume overload has been implicated in RV dilation and dysfunction (4, 5), emerging evidence suggests that aberrant hemodynamic forces—particularly TSS—may drive subclinical myocardial injury long before overt functional decline (6–8). Unlike vascular endothelium, where oscillatory shear stress is well-established in atherosclerosis (9, 10), the cardiac implications of TSS remain poorly understood. Cardiomyocytes are rarely exposed to direct fluid shear stress under physiological conditions due to the protective trabecular architecture and laminar flow patterns (11, 12). However, PR disrupts this equilibrium, generating retrograde diastolic jets that expose RV endocardial surfaces to pathological TSS—a mechanical insult distinct from pressure overload-induced cyclic strain, as the latter primarily results from conditions like RV outflow track (RVOT) obstruction (13–15). This unique hemodynamic milieu may explain why PR patients develop RVOT-predominant fibrosis despite preserved global function (5, 16), a phenomenon inadequately addressed by current guidelines focused solely on volumetric indices.

The NLRP3 inflammasome, a key mediator of sterile inflammation, is activated in cardiomyocytes during ischemia-reperfusion injury and pressure overload cardiomyopathy (17). However, its role in mechanotransduction—particularly in response to direct fluid shear stress—remains unexplored. In vascular endothelium, TSS activates NLRP3 via mechanosensitive ion channels or integrin signaling, promoting cytokine release (e.g., IL-1β, IL-18) and fibroblast activation (18). Crucially, no studies have investigated whether TSS in the RV—particularly in the anatomically vulnerable RVOT—triggers NLRP3-driven inflammation in cardiomyocytes remains unexplored. This knowledge gap hinders development of targeted therapies, as current management remains reactive rather than preventive.

Clinical urgency stems from the unresolved challenges that a significant proportion (30%) of PR patients exhibit rapid RVOT fibrosis progression despite timely pulmonary valve replacement, suggesting irreversible mechanical injury prior to intervention (19). We hypothesize that PR-induced TSS in the RVOT activates the NLRP3 inflammasome in cardiomyocytes, initiating a pro-inflammatory cascade that exacerbates regional fibrosis and global RV dysfunction. To test this, we combined clinical imaging, CFD, and experimental models to dissect the biomechanics-inflammation-fibrosis axis in PR.

## 2 Methods

### 2.1 Participants

This single-center cross-sectional cohort study was approved by the hospital institutional review board (Approve number 2018HXFH021). In total, 6 patients with rTOF and PR (gender 4 M/2 F; age 8–16 years) were included in this study. The exclusion criteria were as follows: (1) trans-annular patch or RVOT patch; (2) residual shunt and residual obstruction examined by echocardiography; (3) residual tricuspid regurgitation (TR); (4) moderate to severe stenosis (trans-annular pressure gradient >40 mmHg) of the RVOT during echocardiography follow-up; (5) patients with inadequate image quality (aliasing, inhomogeneous magnetic field, motion artifact, imaging noising, etc.) for the cardiac function. Patients with trans-annular patches or RVOT patches were excluded to minimize confounding effects from altered RVOT anatomy and hemodynamics (20). TR was excluded to isolate the impact of PR on RV dysfunction (RVD) without additional volume overload from tricuspid valve pathology (21). These criteria aimed to standardize the study population and reduce heterogeneity in hemodynamic and structural variables.

The age at CMR, height, body weight, NYHA classification, and follow-up times after surgery were collected. Patients were examined without contrast agents and informed consent was signed by participant's parents or legal guardians. Written informed consent was obtained from all the participating patients. For patients aged younger than 16 years, written informed consent was obtained from both the parents and the participating patient. CMR protocol and CFD study were provided in Supplementary Materials 1.

### 2.2 Fluid culture of cardiomyocyte

#### 2.2.1 Cell culture

Human immortalized cardiomyocytes (hiCMs) (Meisen Cell Technology Co., Ltd, Zhejiang, China) were cultured in standard DMEM/F-12 medium with 10% fetal bovine serum, 100 U/ml penicillin, and 100 µg/ml streptomycin (all from Solarbio, Beijing, China). The cells were maintained at 37 °C, 95% air, and 5% CO_2_ in a humidified incubator. Cells were passaged every 3 days when reaching 80%–90% confluence using 0.25% trypsin-EDTA (Invitrogen, Carlsbad, CA) and resuspended in fresh DMEM/F-12 medium supplemented with 10% fetal bovine serum, 100 U/ml penicillin, and 100 µg/ml streptomycin. The passaging medium was identical to the standard culture medium to maintain consistency.

#### 2.2.2 Fluid culture

A computerized pump system (Martinsried/Munich, Germany), which includes an Ibidi pump, fluidic unit, and perfusion set (with a length of 15 cm, inner diameter of 1.6 mm, and 10 ml reservoirs), was used for cultivating of hiCMs. The flow shear stress were regulated by the “Pump Control” software. The hiCMs were seeded into the channels of µ-slides I 0.4 mm at a density of 2 × 10^6^ cells/ml medium, and allowed attach to the slide surface for 4 h. The slides were then connected to the perfusion sets, and flow was generated by the Ibidi pump and fluidic unit. Cells were subjected to static, unidirectional or oscillatory flow. Prior to starting the flow culture, adjustments were made to the flow rate and pressure to account for different shear stress. For UF, the flow rate and pressure were default at either 5 dyn/cm^2^ or 15 dyn/cm^2^, depending on the shear stress setting. OF was used to replicate the cardiac cycle in case of pulmonary valve insufficiency. This involved a rapid forward flow of medium for 0.5 s to simulate the heart systole, followed by a quick reverse flow for 0.5 s to mimick heart diastole. The OF parameters were calibrated to mimic the retrograde diastolic flow observed in PR.

Cells were subjected to OF and UF for 4, 8, and 12 h to evaluate acute inflammatory responses. In the control group (static fluid), cells were cultured in µ-slides I 0.4 mm for 4, 8, and 12 h without flow culture. All flow experiments took place in a cell incubator at 37 °C, with 95% air and 5% CO_2_. For protein isolation, cells were cultured in µ-slides I 0.4 mm and pooled from at least 3 slides. Inflammatory factors were isolated from the culture medium in µ-slides I 0.4 mm, with 3 slides pooled together.

### 2.3 Pulmonary regurgitation model

#### 2.3.1 Experimental animals

18 male Sprague-Dawley rats, aged 5 weeks and weighing 140–150 g, were acquired from Chengdu Dashuo Experimental Animal Co., Ltd. in Chengdu, China. Male Sprague-Dawley rats were used to eliminate potential sex-related variability in hormonal profiles and inflammatory responses, as previously described. The Animal Research Ethics Committee of Sichuan University approved the study in accordance with the Guide for the Care and Use of Laboratory Animals.

Rats were put under anesthesia with 5% isoflurane inhalation, and then intubation was done instantly after they were fully anaesthetized, and 2% isoflurane inhalation was used to maintain the anesthesia state. A median sternotomy was performed to execute the suture plication technique. A 7-0 polypropylene suture was threaded through one pulmonary valve sinus and around the hinge points of the pulmonary valve leaflets to induce PR. Transthoracic echocardiography was then used to confirm the successful induction of PR. Only animals with a confirmed PR signal were included in the intervention group. The control group received the same procedure but without creating PR. To prevent infection, Cefazolin (0.2 mg/g) was administered subcutaneously once daily for the first three days post-surgery. Additionally, high-protein feed was provided during the first week post-surgery to aid recovery.

The study evaluated animals in three intervention groups (4, 8, and 12 weeks of follow-up) and a control group (three age- and weight-matched rats per follow-up time point). There are no studies describing the progression of RV remodeling 4–12 weeks after PR induction, but 1 month after PAH induction can lead to both significant RV remodeling (22). Given that the RV is more tolerant to volume overload than to pressure overload, we chose to appropriately extend the time after PR induction to observe the progression of RV remodeling.

CMR cans were performed using a 7.0T MR scanner (BRUKER BIOSPEC 70/30, BRUKER, Ettlingen, Germany), and RV volume data were indexed to body surface area (BSA). RV contraction was assessed using RV ejection fraction (RVEF) and RV peak strain rate (PSR). At the end of the study, all animals were euthanized for histological analysis of the RV. The Meeh-Rubner formula was used to calculate the BSA of the rats:

$$\mathrm{BSA}\;(m^{2})=\left(K\times W^{\frac{3}{2}}\right)/10,000$$

For rats, the constant *K* is 9.1 and *W* is the body weight (g). CMR imaging scan protocol, image post-processing and histologic study were provided in Supplementary Materials 2.

### 2.4 Western blot, ELISA and immunohistochemistry

The assessment of NLRP3 and caspase-1 expression in hiCMs was performed using commercially available NLRP3 (Hu'an biology, China) and caspase-1 (Proteintech, China) western blot kits. The cytokine analysis for levels of IL-1β and IL-18 in culture medium was performed using Human IL-1β and IL-18 ELISA kits (Elabscience). The examine of NLRP3 expression in RV was performed using immunohistochemistry kit (Abcam). All kits were used according to the manufacturer's instructions.

### 2.5 Statistics

IBM SPSS Statistic 26.0 was employed for data analysis. Single-factor repeated design ANOVA was used to compare the repeated measurement data of multiple equidistant time points. One-way ANOVA was used to compare the data of normal distribution. Kruskal–Wallis one-way ANOVA was used to compare the data of non-normal distribution. Non-normally distributed data was subjected to correlation analysis with Spearman correlation. *P* value less than 0.05 was considered to be statistically significant.

## 3 Results

### 3.1 Increased WSS in RVOT caused by PR

**Figure 1:**
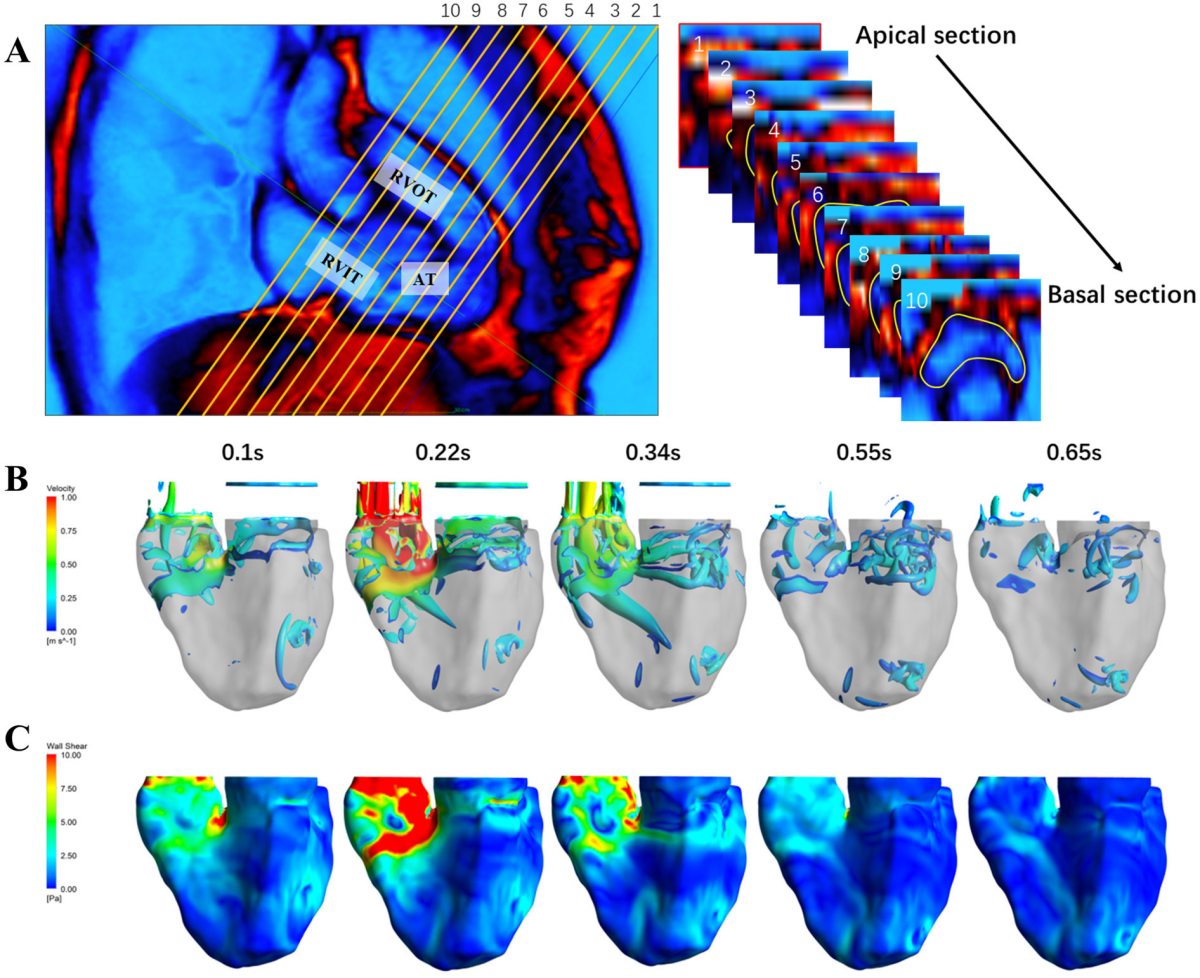
Visualization of RV flow dynamics and TSS during the cardiac cycle. **(A)** The left inset displays a coronal view of the RV include RVIT, AT and RVOT. The right inset illustrates a sequence of transverse section slices from apical to basal region, with yellow lines outlining the endocardium. **(B)** Time-resolved velocity field within the RV at different time points during the cardiac cycle. **(C)** Turbulent shear stress distribution on the RV walls at corresponding time points.

6 patients with rTOF and PR were recruited, the basic data of the patients are shown in Table 1. The coronal plane of RV includes RV inflow track (RVIT), apical trabecular (AT), and RVOT, RV was reconstructed from 10 transverse plane (Figure 1A). The EF of RVIT, AT, and RVOT measured by CMR showed no significant difference among these patients. However, the TKE of RVOT was significantly higher than that of RVIT and AT [RVIT (18.6 ± 4.8) vs. AT (11.9 ± 3.3) vs. RVOT (27.1 ± 8.3), *P* = 0.001] (Table 2). The CFD simulation revealed a high-speed turbulent blood flow in the RVOT (Figure 1B), with the TSS of RVOT consistently higher than that of RVIT and AT (Figure 1C).

**Table 1:** Basic characteristics of patients received CMR scan.

Patient	Sex	Age at TOF repair (month)	Time at CMR scanning			
			Age (year)	Height (cm)	Body weight (kg)	NYHA
1	Male	6	10	147	37	I
2	Male	5	8	130	25	I
3	Female	30	14	156	50	II
4	Female	24	16	167	55	II
5	Male	11	8	135	27	I
6	Male	9	9	135	33	I

**Table 2:** Right ventricle function parameters for each patient.

Patient number	EF (%)			*P* value	TKE (uj/ml)			*P* value
	RVIT	AT	RVOT		RVIT	AT	RVOT	
1	42.1	59.44	50.88		23	13	38	
2	44.4	55.32	60.7		18.6	9.3	27.9	
3	50.38	49.74	53.6		14.1	13.2	22.6	
4	46.1	57.57	59.31		25	17	33	
5	34.7	46.07	52.93		12.5	7.5	14.1	
6	46.7	50.2	46.7		18.6	11.9	27.1	
Mean value	44.1 ± 5.3	53.1 ± 5.1	54.0 ± 5.2	0.07	18.6 ± 4.8	11.9 ± 3.3	27.1 ± 8.3	0.001

RVIT, right ventricle inflow track; AT, apical trabecular; RVOT, right ventricle outflow track; TKE, turbulent kinetic energy.

### 3.2 Oscillatory flow activated NLRP3 inflammasome in cardiomyocytes

**Figure 2:**
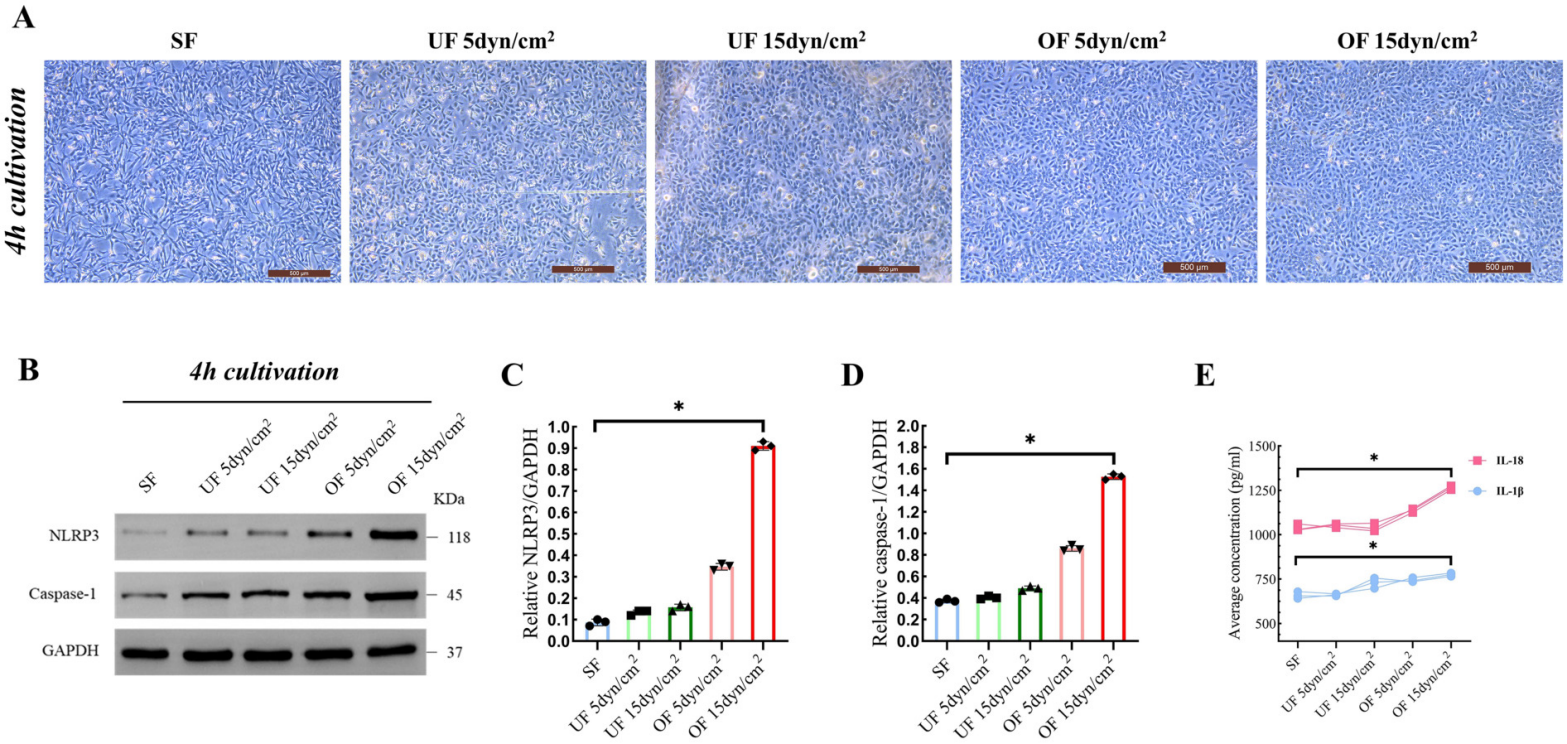
Of activate NLRP3 inflammasome in cardiomyocytes (*n* = 3 per group). **(A)** Representative images of hiCMs after 4 h of cultivation under different shear stress conditions (scale bar: 500 μm). These images illustrate the morphological changes in response to varying shear stress levels. **(B)** Western blot analysis showing the expression levels of NLRP3 and Caspase-1 after 4 h of cultivation under the same shear stress conditions as in **(A) (C,D)** Quantification of NLRP3 and caspase-1 expression relative to GAPDH from the Western blot analysis in **(B) (E)** Average concentrations of IL-18 and IL-1β in the culture medium after 4 h of cultivation under different shear stress conditions. Data are represented as mean ± SEM. Statistical significances were determined by Kruskal–Wallis one-way ANOVA **(C,D**). *P < 0.05.

Morphological observation demonstrated that hiCMs subjected to OF exhibited an elongated spindle-shaped morphology and a disorganized orientation relative to the SF condition (Figure 2A). Representative western blot analyses revealed a significant elevation in the expression levels of NLRP3 and caspase-1 within the OF cohort compared to the SF and UF cohorts (Figure 2B). Quantitative assessments indicated a tenfold upregulation of NLRP3 (0.91 ± 0.02 vs. 0.09 ± 0.01, *P* = 0.01) (Figure 2C) and fourfold upregulation of caspase-1 (1.52 ± 0.03 vs. 0.37 ± 0.02, *P* = 0.012) (Figure 2D) within the OF group in comparison to the SF group. In contrast, the application of UF at both 5 dyn/cm^2^ and 15 dyn/cm^2^ did not yield a significant effect on the expression levels of NLRP3 and caspase-1. Consequently, the concentrations of IL-18 and IL-1β were markedly elevated in the OF group when compared to SF [IL-18: OF 15dyn/cm^2^ (1,264.8 ± 10.7) vs. SF (1,038.6 ± 18.8), *P* = 0.022; IL-1β: OF 15 dyn/cm^2^ (775.1 ± 9.4) vs. SF (658.4 ± 19.6), *P* = 0.03] (Figure 2E).

**Figure 3:**
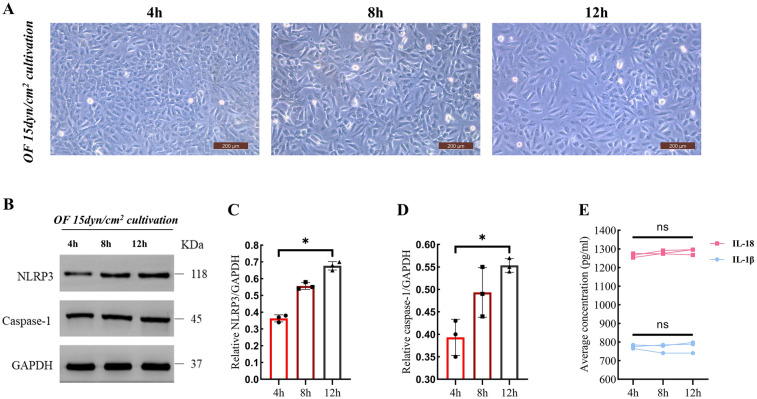
The effects of oF at 15 dyn/cm^2^ on hiCMs morphology and expression levels of NLRP3 and caspase-1 over time. All microscopy images were acquired at identical magnification (20×) to ensure spatial comparability across time points. **(A)** The time-course imaging revealed a spindle-shaped morphology alongside a progressively dispersed cellular distribution (scale bar: 200 μm). **(B)** Western blot bands for NLRP3 and Caspase-1 at 4, 8, and 12 h of OF treat as depicted in **(A) (C,D)** The quantification of NLRP3 and Caspase-1 expression levels, normalized to GAPDH, was derived from the Western blot bands illustrated in **(B)**. **(E)** Average concentrations of IL-18 and IL-1β over 4, 8, and 12 h. Data are represented as mean ± SEM. Statistical significances were determined by single-factor repeated design ANOVA **(C,D**). *P < 0.05.

Time-course imaging showed progressive dispersed cell distribution under OF at a shear stress of 15 dyn/cm^2^, suggesting a loss of cellular clustering capacity (Figure 3A). Representative western blot analyses revealed an elevation in the expression levels of NLRP3 and caspase-1 following 12 h of exposure to OF (Figure 3B). Quantitative assessment indicated a significant increase in the levels of NLRP3 and caspase-1 [NLRP3: 12 h (0.67 ± 0.03) vs. 4 h (0.36 ± 0.02), *P* = 0.022; caspase-1: 12 h (0.55 ± 0.01) vs. 4 h (0.39 ± 0.04), *P* = 0.041] (Figures 3C,D). Despite the presence of IL-18 and IL-1β, their concentrations did not exhibit an increment with the prolonged duration of culture under OF at 15 dyn/cm^2^ (Figure 3E).

### 3.3 PR induced impairment of RV systolic function in rats

**Figure 4:**
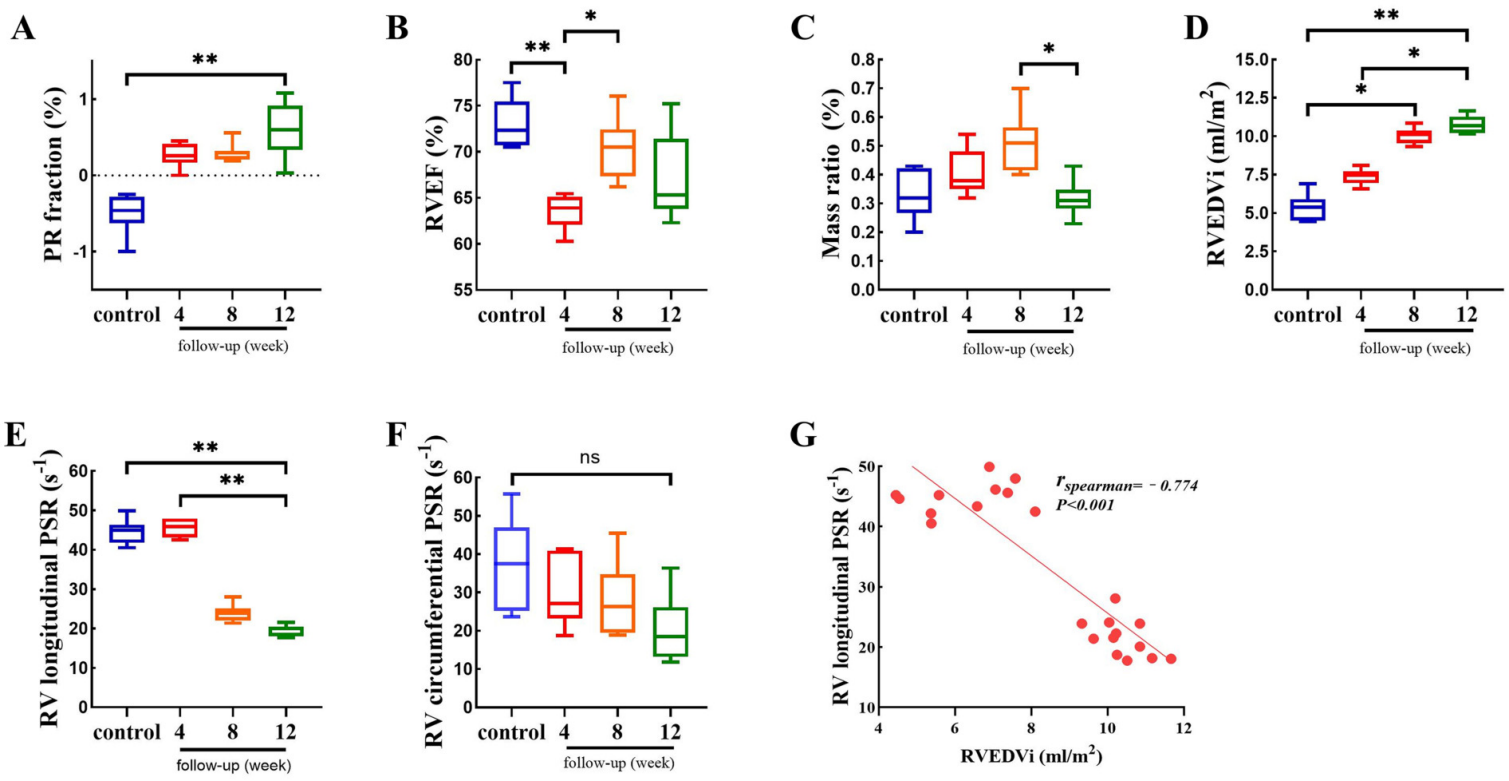
Comparison of RV functional and volumetric para meters between control and intervention groups. Data are represented as mean±SEM. Statistical significances were determined by single-factor repeated design A NOVA **(A-F)** and Spearman correlation **(G)**. *P<0.05, **P< 0.01.

Table 3 summarizes the volumetric and functional parameters of RV. The control data in Table 3 was the average of all time points. The intervention group has a significant PR fraction than the control (12 weeks vs. control, *P* < 0.01), but it was no statistically significant among intervention groups (Figure 4A). The RVEF did not continue to decline although it was significantly varied among all the groups (Figure 4B). The mass ratio was gradually increased until 8 weeks of follow-up, but it was decreased at the 12 weeks follow-up (8 weeks vs. 12 weeks, *P* = 0.019) (Figure 4C). Compared to the control group, RVEDVi increased [4 weeks (7.4 ± 0.4) vs. 8 weeks (10.1 ± 0.5) vs. 12 weeks (10.8 ± 0.6), *P* < 0.01)] (Figure 4D) and RV longitudinal PSR decreased [4 weeks (45.6 ± 2.5) vs. 8 weeks (23.9 ± 2.9) vs. 12 weeks (19.1 ± 0.5), *P* < 0.01)] in the intervention groups (Figure 4E). The RV circumferential PSR exhibit a downward without significant difference between intervention groups (Figure 4F). there was a negative correlation between RVEDVi and RV longitudinal PSR (*r*_spearman_ = −0.774, *P* < 0.01) (Figure 4G).

**Table 3:** CMR parameters of RV function of PR rats.

Functional parameters	Control Mean (95% CI)	Follow-up period (week) Mean (95% CI)			*P* value
		4	8	12	
RVEF (%)	73.0 (70.1, 75.9)	63.5 (61.5, 65.5)	70.3 (66.8, 73.9)	67.1 (62.1, 72.1)	0.032
PR fraction (%)	−49.2 (−77.4, −22.1)	26.5 (9.9, 43.0)	27.8 (13.2, 42.4)	60.3 (22.5, 98.0)	0.009
Mass ratio (%)	33.1 (24.0, 42.2)	40.4 (32.2, 48.7)	51.0 (39.9, 62.1)	31.5 (24.7, 38.3)	0.019
RV longitudinal PSR (s^−1^)	44.6 (41.2, 47.9)	45.6 (43.1, 47.9)	23.9 (21.5, 26.3)	19.1 (17.5, 20.6)	0.001
RV circumferential PSR (s^−1^)	37.4 (24.9, 49.8)	29.9 (20.2, 39.6)	28.0 (17.7, 38.2)	20.2 (10.6, 29.9)	0.165
RVEDVi (ml/m^2^)	5.4 (4.4, 6.3)	7.4 (6.8, 7.9)	10.1 (9.4, 10.6)	10.8 (10.1, 11.4)	0.000
LVEF (%)	71.9 (63.7, 80.2)	67.1 (61.8, 72.5)	69.9 (57.01, 82.7)	61.7 (58.6, 64.7)	0.314

RVEF, right ventricle ejection fraction; Mass ratio, RV mass/LV mass; PSR, peak strain rate; RVEDVi, right ventricle end diastolic volume index; LVEF, left ventricle ejection fraction.

Our animal model demonstrated progressive RV dilation and longitudinal dysfunction within 12 weeks of PR induction, consistent with early adaptive remodeling. However, the absence of significant RVEF decline or circumferential PSR changes may reflect compensatory mechanisms that transiently preserve global systolic function, as observed in the subclinical phase of human PR (23, 24). Notably, human studies indicate that overt RV failure typically manifests over years of chronic volume overload. While our 12-week follow-up captures initial pathological changes, extended observation periods (e.g., 6–12 months) may be required to model the transition from compensatory hypertrophy to decompensated failure. Future studies incorporating longer-term follow-up and longitudinal imaging could elucidate the temporal progression of PR-induced RVD.

### 3.4 PR induced NLRP3 expression in RV

**Figure 5:**
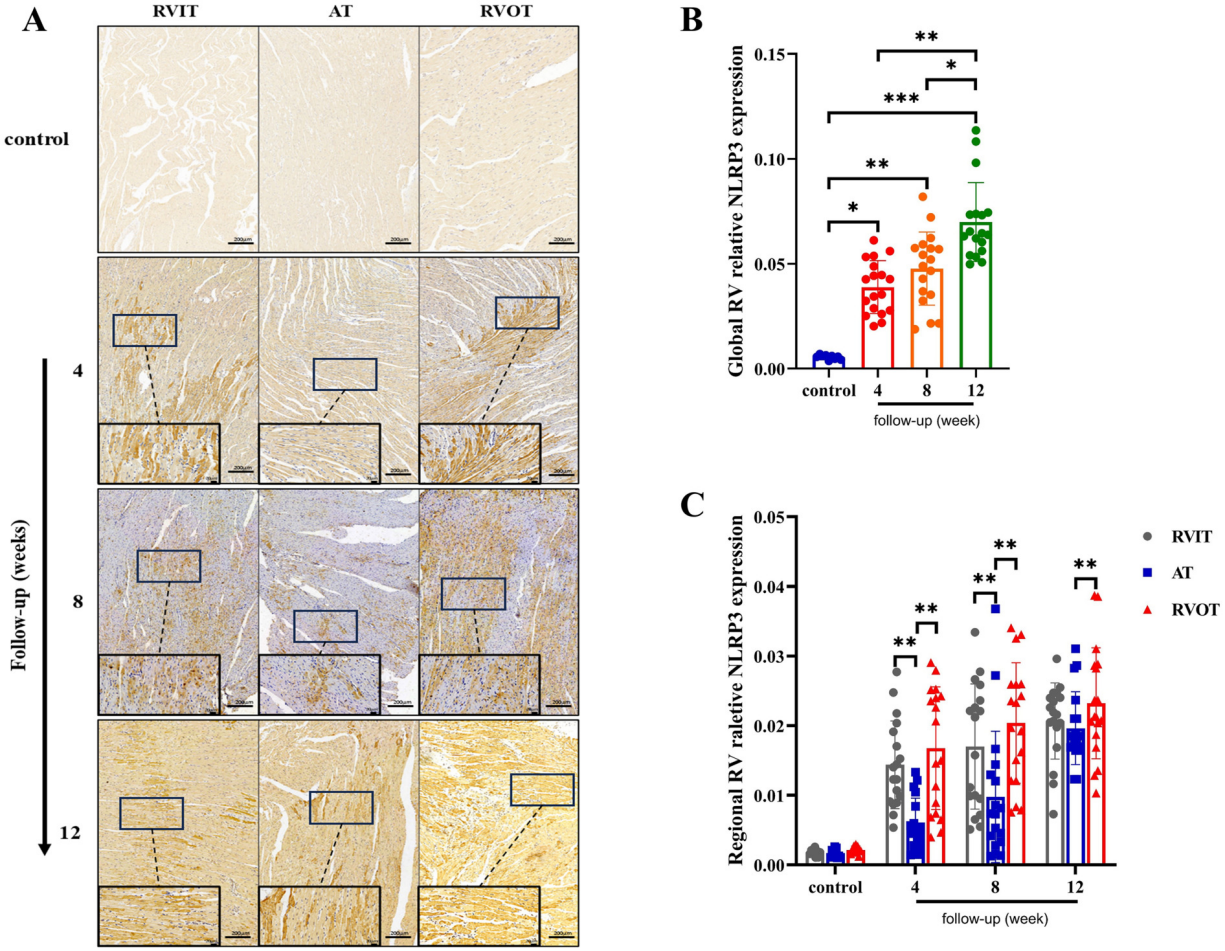
Analysis of RV relative NLRP3 expression at different follow-up time points (*n* = 6 per group). **(A)** Immunohistochemistry stain of NLRP3 in RVIT, AT and RVOT at each follow-up time point; Scale bar: 200 μm and 20 μm. **(B)** Quantification comparison of global RV relative NLRP3 expression in control and intervention groups. **(C)** Quantification comparison of regional RV relative NLRP3 expression in control and intervention groups. Data are represented as mean ± SEM. Statistical significances were determined by single-factor repeated design ANOVA **(B)** and Kruskal–Wallis one-way ANOVA **(C)** *P < 0.05, **P < 0.01, ***P < 0.001.

4 weeks post-surgery, NLRP3 expression was only detected in RVIT and RVOT. By the 8th week, NLRP3 expression had started to appear in AT and had continued to increase in both RVIT and RVOT. At the 12th week, the NLRP3 expression in AT was close to that of RVIT, although both were still lower than that in RVOT (Figure 5A). The global RV NLRP3 expression was higher at 4 weeks and 8 weeks post-surgery compared to the control group [4 weeks (0.04 ± 0.01) vs. control (0.005 ± 0.001), *P* = 0.04; 8 weeks (0.05 ± 0.02) vs. control (0.005 ± 0.001), *P* = 0.001], but the difference was not significant between the two time points (Figure 5B). The NLRP3 expression was significantly higher at 12 weeks post-surgery compared to the previous two time points [12 weeks (0.07 ± 0.02) vs. control (0.005 ± 0.001), *P* < 0.001; 12 weeks (0.07 ± 0.02) vs. 4 weeks (0.04 ± 0.01), *P* < 0.01; 12 weeks (0.07 ± 0.02) vs. 8 weeks (0.05 ± 0.02), *P* = 0.039] (Figure 5B). The regional RV NRLP3 expression was notably distinct from each other, with the NLRP3 expression in RVOT being always higher than that in RVIT and AT at each follow-up time point (Figure 5C).

### 3.5 PR induced inflammatory cell infiltration in RV

**Figure 6:**
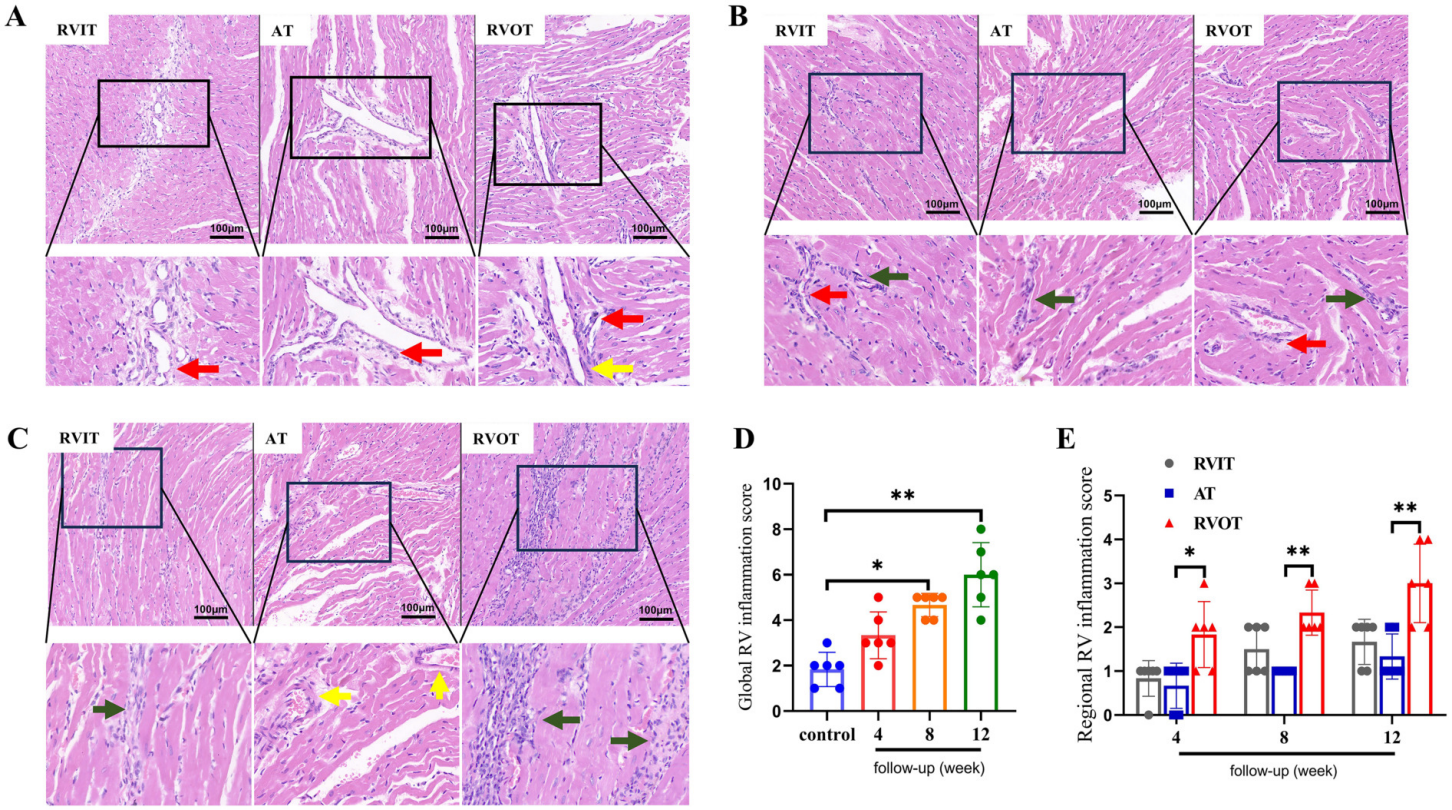
Analysis of myocardial inflammation of RV at different follow-up time points via H-E stain (*n* = 6 per group). **(A)** 4 weeks after PR surgery. **(B)** 8 weeks after PR surgery. **(C)** 12 weeks after PR surgery. Scale bar: **A, B, C**, 100 μm. **(D)** Comparison of inflammation scores of global RV. **(E)** Comparison of inflammation scores of regional RV in intervention groups. Data are represented as mean ± SEM. Statistical significances were determined by single-factor repeated design ANOVA **(D)** and Kruskal–Wallis one-way ANOVA **(E)** *P < 0.05, **P < 0.01. ***Red arrow***: peri-microvascular inflammatory cells; ***Yellow arrow***: wall thickening of microvessels; ***Green arrow***: inflammatory cells in extracellular matrix.

4 weeks post-surgery, the RVIT and AT showed the presence of peri-microvascular inflammatory cells, with the RVOT also exhibiting thickening of microvessel walls (Figure 6A). By the 8 weeks post-surgery, the inflammatory cells had migrated from the microvessels to the perivascular region, forming inflammatory lesion in the extracellular matrix (Figure 6B). At 12 weeks post-surgery, both the RVIT and AT exhibited thicker microvascular walls increased inflammatory cell presence, with a significant accumulation of inflammatory cells in the RVOT extracellular matrix, leading to extensive inflammatory lesions (Figure 6C). The comparison of global RV [8 weeks (4.7 ± 0.5) vs. control (1.8 ± 0.7), *P* = 0.022; 12 weeks (6.0 ± 1.4) vs. control (1.8 ± 0.7), *P* < 0.01] and regional RV inflammatory injury was semi-quantitatively as inflammation score (Figures 6D–E).

### 3.6 Fibrosis correlates with RV remodeling and systolic dysfunction

**Figure 7:**
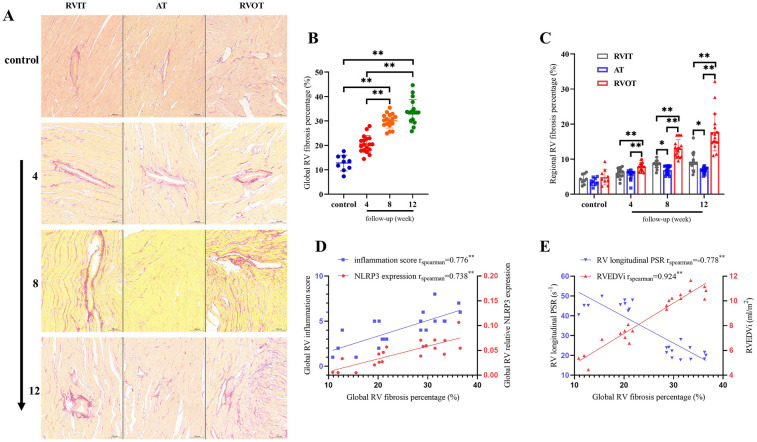
Fibrosis correlates with RV remodeling and systolic dysfunction (*n* = 6). **(A)** Comparison of fibrosis in the three regions of RV in control and intervention groups. **(B)** Quantitative analysis global RV fibrosis in control and intervention groups. **(C)** Quantitative analysis of regional RV fibrosis in control and intervention groups. **(D)** Correlation between the global RV fibrosis percentage and RV inflammation score with NLRP3 expression. **(E)** Correlation between the global RV fibrosis percentage and RVEDVi with RV longitudinal PSR. Statistical significances were determined by single-factor repeated design ANOVA **(B)** and Kruskal–Wallis one-way ANOVA **(C)** *P < 0.05, **P < 0.01.

Histological analysis revealed that PR exacerbated RV fibrosis progressively, with significant differences in the global RV fibrosis percentage at different time points[12 weeks (33.9 ± 4.8) vs. 8 weeks (30.0 ± 2.9) vs. 4 weeks (20.5 ± 3.5) vs. control (12.8 ± 3.2), *P* < 0.01] (Figures 7A,B). Regional disparities in RV fibrosis were evident, with RVOT fibrosis being predominant (Figure 7C). Furthermore, global RV relative NLRP3 expression (*r*_spearman_ = 0.738, *P* < 0.01) and inflammation score (*r*_spearman_ = 0.776, *P* < 0.01) demonstrated positive correlations with global RV fibrosis percentage (Figure 7D). This suggests that inflammation is a contributing factor to RV fibrosis. Additionally, a strong positive correlation was observed between global RV fibrosis percentage and RVEDVi (*r*_spearman_ = 0.924, *P* < 0.01), while a negative correlation was found with RV longitudinal PSR (*r*_spearman_ = −0.778, *P* < 0.01) (Figure 7E). These findings imply that the fibrotic process adversely affects RV expansion and contraction, with the RVOT identified as a principal lesion contributing to overall RVD.

## 4 Discussion

Our findings demonstrate that PR-induced TSS activates the NLRP3 inflammasome in cardiomyocytes, leading to cytokine release (IL-1β, IL-18) and progressive RV fibrosis. This biomechanics-inflammation-fibrosis axis is particularly pronounced in the RVOT, where TSS levels are highest, suggesting a direct spatial correlation between hemodynamic stress and pathological remodeling. The temporal progression—early NLRP3 activation (4–12 h *in vitro*) preceding fibrosis (4–12 weeks *in vivo*)—supports a chronological sequence where inflammation initiates structural damage. These observations align with studies linking oscillatory shear stress to endothelial NLRP3 activation in atherosclerosis (18, 25), yet extend this paradigm to cardiomyocytes in volume-overload cardiomyopathy.

This study suggests that TSS may serve as a novel noninvasive biomarker for assessing subclinical right ventricular injury in patients with PR. Current guidelines mainly rely on RVEF and volumetric indices to determine the timing of surgery (26), but there is a lag in these indices. Quantification of the TSS of RVOT (Figure 1) by advanced imaging techniques such as 4D-flow CMR and CFD (27) may identify high-risk patients in need of early intervention.

Studying the effects of TSS on cardiomyocytes in humans is extremely challenging. The heart's constant contraction and complex blood flow create a dynamic environment that makes it nearly impossible to directly measure and manipulate TSS within a living human heart. Recreating the biomechanical environment of the heart in a laboratory setting is also very difficult. Cardiomyocytes are subjected to various mechanical forces, such as shear stress from blood flow and strain from heart contractions (28). Developing an *in vitro* system that can accurately mimic these conditions is a complex task. Some progress has been made using flow chambers and other devices to simulate shear stress, but these systems are unable to fully replicate the multifaceted biomechanical environment of the heart (29). These challenges highlight the complexity of understanding the specific role of TSS in cardiomyocytes function.

Previous studies have demonstrated that oscillatory shear stress is more inflammatory compared to laminar shear stress (30). Our *in vitro* experiments demonstrated that oscillatory flow, rather than unidirectional flow, triggers an inflammatory response in cardiomyocytes through activation of NLRP3 inflammasome (Figures 2, 3), which differs significantly from the mechanism of vascular endothelial cell response to TSS, which engage in a broader range of mechanotransduction pathways that do not predominantly involve the NLRP3 inflammasome (31, 32). Notably, the anatomical structure of the RVOT, as the main power zone of RV ejection (e.g., sparse trabeculation and small radius of curvature), may lead to a greater susceptibility to turbulence in this region (33, 34), which in turn produces a persistent high TSS (Figure 1). This region-specific mechanical abnormality may explain the high prevalence of RVOT fibrosis (Figure 7C) in the clinic (35, 36).

While prior studies focused on fibrosis as an endpoint, our data reveal NLRP3 activation as an upstream event. It is well known that NLRP3 inflammasome plays a key role in the inflammatory response in various cardiovascular diseases (37–39). Our animal experiments revealed that TSS can activate NLRP3 inflammasome in cardiomyocytes, revealing a novel link between biomechanical forces and cardiac inflammatory responses. While fibrosis is a hallmark of end-stage RV stiffness, our findings highlight NLRP3-mediated inflammation as a potential initiating mechanism. Inflammatory cytokines (e.g., IL-1β, IL-18) released from NLRP3-activated cardiomyocytes can drive fibroblast proliferation and collagen synthesis, ultimately leading to perivascular and interstitial fibrosis (40). Our observation of simultaneous upregulation of NLRP3 (Figure 5) and fibrosis progression associated with increased RVEDVi and decreased longitudinal PSR in RV (Figure 7) supports our view, suggesting that inflammation precedes structural remodeling chronologically. The regional predominance of fibrosis in the RVOT—a site of maximal TSS—further supports the biomechanical-inflammatory-fibrotic axis. While fibrosis is a terminal outcome, targeting NLRP3 may offer therapeutic potential to interrupt this cascade.

The present study found a significant correlation between the RV fibrosis and RVEDVi and systolic function (longitudinal PSR) (Figure 7E), which is align with the conventional view that PR leads to RVD mainly from volume overload. But our study also reveals a new pathway of biomechanics-inflammation-fibrosis underlying RVD. While the majority of cardiomyocytes are not directly exposed to fluid shear stress, endocardial mechanotransduction may propagate inflammatory signals to deeper myocardial layers via paracrine pathways or intercellular communication, thereby affecting neighboring cardiac fibroblasts and cardiomyocytes and regulating myocardial remodeling (28). Although our model focuses on the direct effects of oscillatory shear stress on cardiomyocytes, we acknowledge that *in vivo* pathology involves a complex interplay of hemodynamic forces, neurohormonal factors and cell-cell interactions. This axis of pathology may explain why some patients still develop progressive RV failure after PVR: even after volume loading is lifted, the TSS-induced inflammatory microenvironment has initiated an irreversible fibrotic process. Therefore, future studies should integrate endothelial cells, cardiomyocytes and fibroblasts to construct a 3D co-culture systems, which like the cardiac organism on a chip, could better reproduce the direct effect of cardiomyocytes on oscillatory shear stress.

In general, this study linking hemodynamic abnormalities to molecular pathways, we provide a framework for early diagnosis (TSS quantification) and targeted therapy (NLRP3 inhibition). These advances challenge the traditional view of PR as a purely volume-overload pathology, positioning biomechanical stress as a therapeutic frontier in congenital heart disease.

### 4.1 Limitations

Firstly, the exclusion criteria may restrict the applicability of our results to a wider rTOF patient population. Subsequent investigations should encompass patients with trans-annular patches, RVOT patches, and diverse TR levels to corroborate these findings in more intricate clinical contexts. The limited human sample size and focus on adolescents may have obscured the variability of TSS in adult PR patients, necessitating future age-stratified cohort expansions for validation.

Notably, human studies indicate that overt RV failure typically manifests over years of chronic volume overload. While our 12-week follow-up captures initial pathological changes, extended observation periods (e.g., 6–12 months) are required to model the transition from compensatory hypertrophy to decompensated failure. Our study's exclusive use of male rats aimed to mitigate sex-specific variability, albeit at the expense of findings' applicability to females. The unavailability of 4D-flow sequences in 7.0T CMR that can be used in rodents precluded precise quantification of RV blood flow patterns, particularly TKE and shear stress gradients. This technical constraint may account for discrepancies in flow characterization between rodent models and human CMR data.

We could not assess hemodynamic parameters between humans and rats due to limitations in animal CMR technology. Furthermore, the activation mechanisms of the NLRP3 inflammasome in cardiomyocytes under fluid culture require further clarification, and the rescue mechanisms of RV function via NLRP3 inhibitors necessitate additional exploration.

## 5 Conclusion

This study demonstrates that PR-induced turbulent shear stress in the RV outflow tract activates NLRP3 inflammasome in cardiomyocytes, initiating a pro-inflammatory cascade that drives regional fibrosis and functional decline. Quantifying TSS via advanced imaging may serve as an early biomarker for subclinical RV injury, while targeting NLRP3 signaling could offer a novel therapeutic strategy to mitigate fibrosis in PR patients.

## Data Availability

The original contributions presented in the study are included in the article/Supplementary Material, further inquiries can be directed to the corresponding author.
