# Supplementary material for: NLRP3 inflammasome activation by turbulent shear stress drives right ventricular outflow tract fibrosis in pulmonary regurgitation
Source: Front Cardiovasc Med. 2025 May 2;12:1546581. doi: 10.3389/fcvm.2025.1546581 (PMC12081451; doi:10.3389/fcvm.2025.1546581)
Supplement: Supplementary file 1 [file Datasheet1.docx]

***CMR protocol and CFD study***

All subjects underwent 2D fast imaging employing steady-state acquisition (2D FIESTA) cine sequence performed with a 3.0T scanner (General Electric, Signa, Wisconsin, USA) by long and short axis view covering the RV to measure cardiac function. The Black blood steady-state free precession (SSFP) sequence is combined with T2-weighted imaging to enhance the contrast between the vascular wall and the blood flow in the vascular lumen. The following MR imaging parameters were used: repetition time, 2.5-5ms; echo time, 1-3ms; acquisition matrix, 256×256; flip angle, 60°; interslice gap, 0mm; section thickness, 8mm. Finally, 25 phases per cardiac cycle were reconstructed.

4D flow CMR was used to characterize and quantify flow hemodynamics in the whole heart. Patients lay supine with an 8-channel phased array coil covering the chest, ensuring complete coverage of the heart and chest vessels. Electrodes for magnetic resonance electrocardiogram (ECG) monitoring were attached to the chest, enabling ECG and respiratory gating. Radial sampling with 5-point balanced velocity coding was employed for the 4D-flow CMR sequence. The scan volume included the entire thoracic aorta and pulmonary artery. Phase-contrast sequences were used to obtain the three-dimensional velocity field of blood flow. Sequence parameters were as follows: echo time, 1.9-3.7ms; repetition time, 4.2-6.4ms; flip angle 8–12°; field of view of 400x400x400mm; voxel size, scanning matrix of 2x2x2mm^3^ voxel size; temporal resolution, 30-50ms; velocity sensitivity, 150-250cm/s (based on the aortic valve velocity measured by echocardiography); parallel imaging with reduction factor, R=2. The resulting scan time was on the order of 30-40 minutes.

The tricuspid valve and pulmonary artery flow velocity, along with RV chamber size measured by CMR, were inputted into Ansys Fluent software for CFD simulation to calculate the spatial averaged WSS (SAWSS) and time averaged WSS (TAWSS).

$$SAWSS=\frac{1}{S}\int_{0}^{S} \left| \tau\left( s,t \right) \right|ds$$

$$TAWSS=\frac{1}{T}\int_{0}^{T} \left| \tau\left( s,t \right) \right|dt$$

where S and T are the area of surface wall and the cardiac period.
