# Supplementary material for: NLRP3 inflammasome activation by turbulent shear stress drives right ventricular outflow tract fibrosis in pulmonary regurgitation
Source: Front Cardiovasc Med. 2025 May 2;12:1546581. doi: 10.3389/fcvm.2025.1546581 (PMC12081451; doi:10.3389/fcvm.2025.1546581)
Supplement: Supplementary file 2 [file Datasheet2.docx]

***Cardiac MRI scan protocol***

A 7.0T animal MR scanner was used to conduct a CMR. A radiofrequency signal transmitting coil (inner diameter of 72mm) and an array surface echo signal receiving coil (inner diameter of 56mm) were employed. Anesthesia was maintained with 2.5% isoﬂurane for the during scanning. With temperature, respiratory, and heart rate monitoring, rats were placed on the scanning bed. The electrocardiogram gating has been adjusted to a range of 350-400bpm, thus limiting the occurrence of artifacts by only collecting data when the rat's heart rate is in this range. The scanning sequence was FLASH-cine and T2 mapping.

T2 Mapping Protocol:

-repetition time: 8.0ms

-echo time: 2.5ms

-flip angle: 15°

-field of view: 50mm×50mm

-image matrix size:256×256

-slice thickness: 1.5mm

-slice gap: 0mm

-cardiac trigger: end-diastolic phase, 22-30 phases per cardiac cycle

-5 minutes per slice.

The scanning included RV continuous two-chamber and four-chamber views in short axis and long axis.

***Image post-processing***

CVI 5.9 software (Circle Cardiovascular Imaging, Calgary, AB, Canada) was used to import CMR images of RV for cardiac function and strain analysis. The RV volume was determined by delineating the endocardium, including the papillary muscle, in the blood pool at end diastole and end systole, as well as the RVOT. The continuous short and long axis images were added to the tissue tracking module for strain analysis. In this study, 2D circumferential and longitudinal strain were obtained. PR degree was assessed by calculating the regurgitation fraction defined as (RVSV–LVSV)/RVSV; RVSV and LVSV being the RV and LV stroke volumes. The remodeling of RV was assessed by calculating the ventricular mass ratio defined as RV mass/LV mass at diastole.

***Histologic Study***

The RV myocardium was divided into three sections: RVIT, AT, and RVOT. The RVIT and RVOT are divided by the supraventricular crest, with the AT located below the attachment point of the tricuspid papillary muscle.

Samples were fixed in 4% formalin before being embedded in paraffin. Each part was then sliced in 3μm with 2 slices per slide. Slices were stained with Sirius red and hematoxylin-eosin. The slides were then examined under an orthostatic optical microscope (Nikon, Japan). All slides were scanned in the slide scanner (Pannoramic MIDI, 3DHISTECH) for further histological examination. The heart tissue slides were viewed by using SlideViewer (3DHISTECH) at 10× and 40× magnification.

The inflammation score is a reflection of myocardial injuries using a semi-quantitative scale from 0 to 4, as follows: 0 = no injury, 1 = isolated myocyte injury, 2 = one focal area of injury, 3 = two or more areas of injury, and 4 = diffuse areas of damage compromising more than 50% of the myocardium. The inflammation score was calculated using the entire slice of each part of RV, with the damage limited to the myocardium and endocardium, excluding the epicardium.

The fibrosis was measured using Sirius red stain in 3 randomly selected fields per slice. The percentage of fibrotic area to the total area was used to determine the extent of fibrosis. ImageJ 6.0 (Media Cybernetics, Inc., Rockville, MD, USA) was used for image analysis. Slides and histology analysis were both done by the same person blinded to the group information.

**
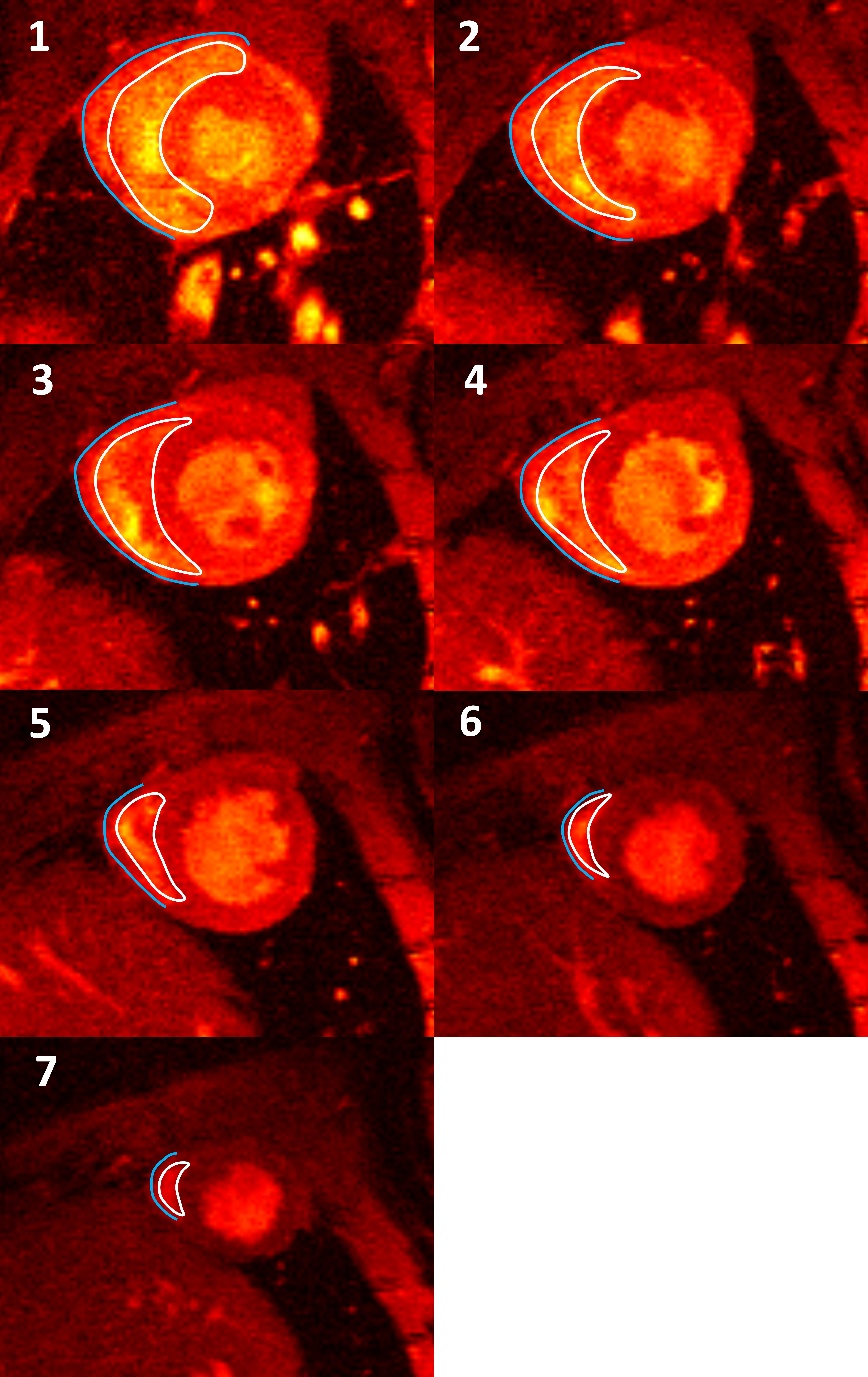
Supplementary Figure**: The transverse section of right ventricle in PR rats, from basal (1) to apex (7). White line: endocardium, blue line: epicardium.
